# The Influence of a Shoe’s Heel-Toe Drop on Gait Parameters during the Third Trimester of Pregnancy

**DOI:** 10.3390/bioengineering9060241

**Published:** 2022-05-31

**Authors:** Xin Li, Zhenghui Lu, Dong Sun, Rongrong Xuan, Zhiyi Zheng, Yaodong Gu

**Affiliations:** 1 Faculty of Sports Science, Ningbo University, Ningbo 315211, China; 2011042028@nbu.edu.cn (X.L.); 2011042030@nbu.edu.cn (Z.L.); sundong@nbu.edu.cn (D.S.); 2 The Affiliated Hospital of Medical School, Ningbo University, Ningbo 315211, China; fyxuanrongrong@nbu.edu.cn; 3 ANTA Sports Science Laboratory, ANTA (China) Co., Ltd., Xiamen 361008, China

**Keywords:** negative heel shoes, positive heel shoes, gait, pregnant women, OpenSim, IDEEA

## Abstract

Background: Changes in physical shape and body mass during pregnancy may increase the risk of walking falls. Shoes can protect and enhance the inherent function of the foot, helping to maintain dynamic and static stability. Methods: Sixteen women during the third trimester of pregnancy participated in this study to investigate the effect of negative heel shoes (NHS), positive heel shoes (PHS), and normal shoes (NS) on spatiotemporal parameters, ground reaction force (GRF), and stability. Differences in spatiotemporal parameter, GRF, and center of pressure (COP) between footwear conditions were examined using Statistical Parametric Mapping (SPM) and repeated measures analyses of variance (ANOVA). Results: The walking speed and step length increased with the increase in heel-toe drop. The anterior-posterior (AP)-COP in NHS decreased significantly (*p* < 0.001). When wearing NHS, peak posterior angles were significantly lower than NS and PHS (*p* < 0.05). Conclusions: The results show that changing the heel-toe drop can significantly affect the gait pattern of pregnant women. Understanding the gait patterns of pregnant women wearing shoes with different heel-toe drops is very important for reducing the risk of injury and equipment design.

## 1. Introduction

Pregnancy induces tremendous changes in the body to accommodate a growing fetus [1]. During pregnancy, hormonal, anatomical, and physiological changes occur in the female body. These changes due to pregnancy include mass redistribution, an anterior shift in the center of gravity location, and increased joint and ligament flexibility [2,3,4,5,6]. These changes during pregnancy can cause physical pain and an increased risk of falls, especially in the third trimester [4,7]. During pregnancy, nearly a quarter of employed women sustain a fall [8]. This fall may result in musculoskeletal injury and maternal or fetal death [9,10,11].

Walking is the most commonly chosen type of physical activity during pregnancy [12]. The gait parameters, balance, and center of mass of pregnant women changes during walking and leads to a higher risk of falling [1,6,13,14]. The rate of falls during pregnancy is similar to that of women over 65 [6,8]. A decreased step length and cadence, increased base of support, and longer double support time are seen with the progression of pregnancy [15,16]; these changes provide a safer and more exploratory way for pregnant women to walk. However, results point toward excessive deviations from the optimal habitual spatiotemporal gait pattern as a pivotal factor that may contribute to falls in pregnant women [16]. Mei et al. studied pregnant women’s gait biomechanics, which revealed lower limb kinematic and foot pressure alterations, and found that mean pressure in the forefoot increased. The center of pressure (COP) trajectory highlights a fall risk, particularly in the third trimester [4]. To improve their walking stability, pregnant women often use specially designed products, such as daily wearing shoes.

Shoes can protect and enhance the inherent function of the foot, helping to maintain dynamic and static stability [17,18]. Many previous studies have focused on changing the shape and materials of a shoe sole to reduce pregnant women’s foot discomfort [19,20]. Jang et al. designed balanced incline shoes [21] and reported that the balanced incline shoes corrected the postures and stabilized the gait pattern. 

Research about the effects of different heel-toe drop shoes on pregnant gait parameters is lacking. Heel-toe drop is the height difference between the heel and the forefoot of the shoe [22]. In positive heel shoes (PHS; Table 1 includes a description of abbreviations and acronyms used), the heel is higher than the toe part. In contrast, in the negative heel shoe (NHS), the toe part is higher than the heel [23]. Advocates of shoes with negative inclination believe that negative heel inclination decreases lumbar lordosis, causing the center of gravity to shift backwards [24,25]. As a result, back and hip pain can noticeably be reduced [19]. However, the American College of Obstetricians and Gynecologists (ACOG) recommends wearing positive heel shoes (PHS) to relieve back pain during pregnancy [26]. However, few studies have investigated the effect of different heel-toe drops on spatiotemporal parameters, ground reaction force (GRF), and the dynamic balance in the third trimester of pregnancy. It is necessary to know the effects of the different heel-toe drops to design maternity shoes and keep pregnant women healthy.

This study aimed to use a musculoskeletal simulation and Statistical Parametric Mapping (SPM)-based approach to investigate the effect of different heel-toe drops (negative 1.5 cm, 0 cm, positive 1.5 cm) on the spatiotemporal parameter, GRF, and dynamic balance during the third trimester of pregnancy. The results can provide a theoretical basis and ideas for the design of shoes for pregnant women in the third trimester.

## 2. Materials and Methods

### 2.1. Participants 

Sixteen healthy third-trimester primigravid pregnant women (age: 28.4 ± 2.30 years and height: 1.63 ± 0.04 m and trimester: 33.43 ± 3.37 w) participated in the study. Exclusion criteria included the following medical conditions: lupus, rheumatoid arthritis, gestational diabetes mellitus, hypertension, musculoskeletal or neurologic abnormalities, and any other conditions affecting postural stability [5]. All participants understood the purpose and significance of the research and signed an informed consent form. This study with detailed guidelines for participants’ safety and experiment protocols was approved by the Human Ethics Committee of Ningbo University.

### 2.2. Shoe Conditions 

**Figure 1:**
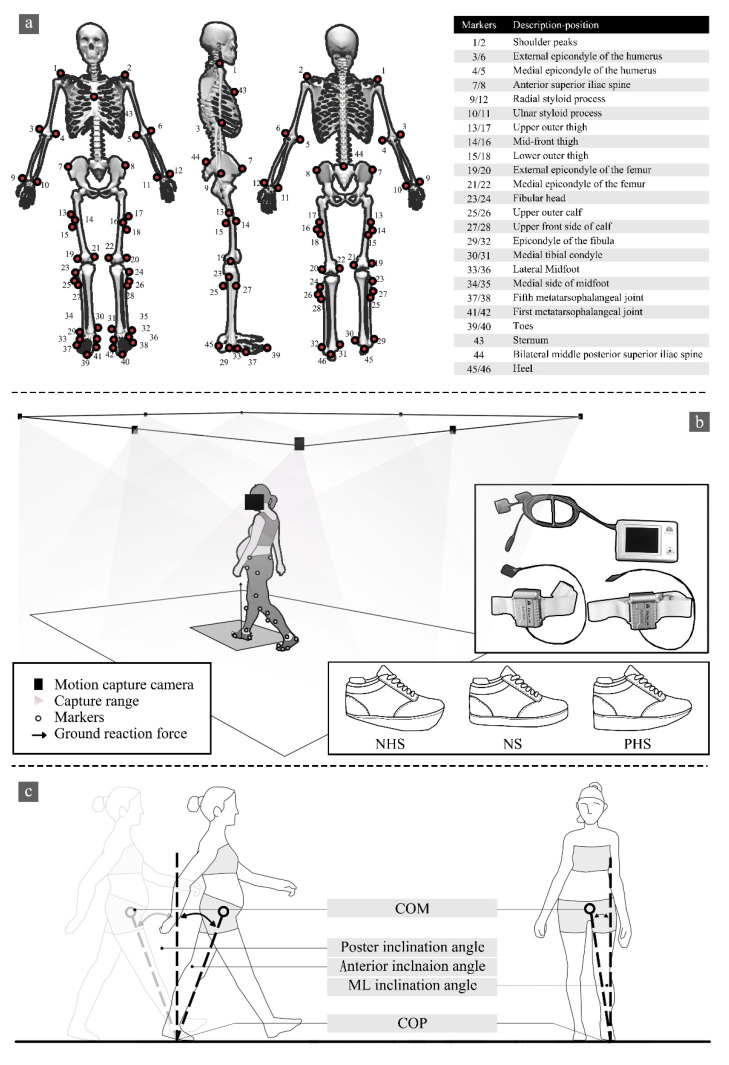
(**a**) Marking point paste location. (**b**) Experimental process, IDEEA position, and shoe conditions between NHS (negative 1.5 cm drop), NS (no drop), and PHS (1.5 cm drop). (**c**) Diagrammatic illustration of COM-COP inclination angles.

All participants conducted this study in shoes with a NHS, normal shoes (NS), and PHS (Figure 1b). The NS were commercially available walking shoes. The NHS and the PHS were self-fabricated based on the NS in our laboratory. For the three conditions, the shoes were identical models and designs in the upper and outsole. 

### 2.3. Testing Procedure 

All participants walked with IDEEA (IDEEA, MiniSun, Fresno, CA, USA) on a 6.5 m walkway at their self-selected comfortable speed to present normal gait characters, striking their right foot on the force plate. Sensors were connected to a 32 Hz main recorder. Previous studies have shown the reliability of IDEEA in measuring gait parameters [27,28,29]. Each footwear condition was collected with three successful trials for analysis. At the same time, an eight-camera Vicon motion capture system (Vicon Metrics Ltd., Oxford, UK) was used to capture the motion trajectory. The embedded AMTI force plates (AMTI, Watertown, MA, USA) recorded the GRF synchronously, with 200 Hz and 1000 Hz, respectively, as shown in Figure 1b. The camera system was calibrated to residual errors of 2.5 mm over a recording volume of approximately 6.5 m × 1.5 m × 1.80 m (L × W × H). The force plate was embedded in the middle of a 6.5-m walkway and covered with floor tiles to minimize participants’ awareness of its presence. The original gait-2392 model in OpenSim was used for this study, with 23 degrees of freedom and 92 muscles (Figure 1a) [30].

### 2.4. Data Processing

Gait analyses were performed using a wearable intelligent analyzer (IDEEA, MiniSun, Fresno, CA, USA) equipped with accelerometers and gyroscopes, as shown in Figure 1b. The wearable intelligent analyzer consists of the main recorder and two secondary recorders. The gait data were collected and transmitted to the main recorder by the sensor affixed to the subject; each accelerometer used a proprietary algorithm [31]. The IDEEA was easy to wear and had almost no interference with normal walking. After the data acquisition was completed, the data were saved in the main recorder and downloaded to the computer. IDEEA Version 3.01 (IDEEA3, MiniSun, Fresno, CA, USA) was used for analysis [27]. The software equipped with the equipment can intercept the range of gait data needed and process it, and directly output walking speed, step frequency, stride length, and support time.

According to Winter’s [32] description of the selected frequency for filtering biomechanical signals, the residual data analysis was carried out in subsets to determine the most appropriate signal-to-noise ratio. Marker trajectories and ground reaction forces were filtered by a zero-delay fourth-order Butterworth low-pass filter at 12 Hz and 30 Hz. A threshold of 20 N on the vertical GRF was applied to identify the initial foot contact and toe-off [33]. The magnitudes of each GRF component were normalized to the percentage of the participant’s body weight, and the stance phase of each participant was normalized to 100% of their stance phase’s duration [34]. The musculoskeletal model used was the generic OpenSim model Gait 2392 (Figure 1a), which has 23 degrees of freedom and 92 muscles [30] and calculates the center of mass (COM) in OpenSim.

### 2.5. Outcome Measures

The parameters evaluated in the study were: (1) Walking speed (m/s): the distance walked along the walkway per second. (2) Step frequency (steps/min): the number of steps per minute. (3) Stride length (m): the distance from one heel to the same heel touching the ground again during walking. (4) Double support time/single support time (%): double support time refers to the time taken by the use of biped support in a gait cycle, and single support time refers to the time spent using single foot support in a gait cycle. Double support time/single support time reflects the stability of the participants when walking, where the lower the ratio, the better the stability of the participants [27]. (5) Three-dimensional ground reaction forces (3D-GRF): GRF supports the body against gravity and accelerates the center of mass during walking. GRF is included in the vertical, anterior–posterior, and medial–lateral directions recorded from a three-dimensional force plate [35,36]. (6) The range of COP motion, including the medial–lateral range of the COP (ML-COP) and anterior–posterior range of the COP (AP-COP), were derived and averaged for all participants. (7) Center of mass (COM) and center of pressure (COP) inclination angles: we defined COM-COP inclination angles as the angle formed by the intersection of the line connecting the COP and COM with a vertical line through the COP [37], as shown in Figure 1c.

### 2.6. Data Analysis

Statistical analyses were performed using SPSS 16.0 (SPSS, Chicago, IL, USA) statistical analysis software. One-way repeated-measures analysis of variance (ANOVA) was performed to analyze the effects of different conditions on spatiotemporal parameters and peak COM-COP inclination angles. In the event of a significant main effect, post-hoc pairwise comparisons were conducted on all significant main effects, using a Bonferroni adjustment. Statistical parametric mapping based on the SPM1D package for Matlab (Mathworks, Natick, MA, USA) was used to compare the 3D GRF and COP statistically. In agreement with Patakt et al., SPM was implemented hierarchically, analogous to one-way repeated measures ANOVA (SPM F) with a post-hoc paired t-test [38]. The conditions NS vs. NHS, NS vs. PHS, and PHS vs. NHS were chosen to compare the 3D-GRF and COP waveforms [39,40]. The significance level was set at 0.05.

## 3. Results

### 3.1. Gait Spatiotemporal Parameters

Significant main effects were found for stride length and walking speed (Table 2). Post-hoc tests revealed significantly higher stride length for PHS compared with NHS. Furthermore, post-hoc tests revealed significantly lower walking speed for NHS when compared with NS and PHS. No significant differences were found in step frequency and double support time/single support time.

### 3.2. GRF

**Figure 2:**
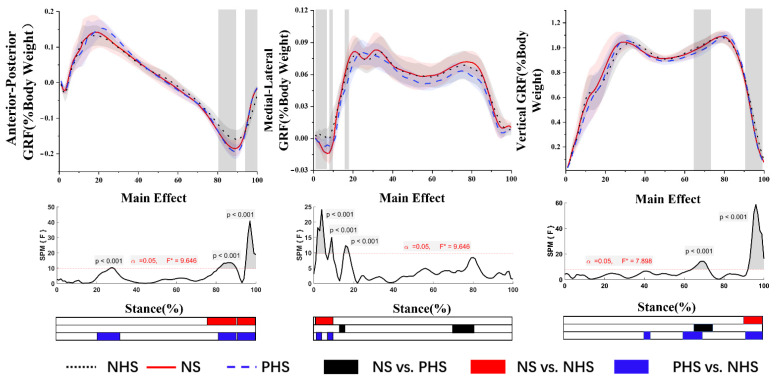
Ground reaction forces in anterior–posterior, medial–lateral and vertical directions (mean and SD) between NHS, NS, and PHS. The grey areas indicate significant differences between conditions, followed by the time-dependent F-values of the SPM. Colored bars beneath each plot indicate significant differences between waveforms, whereas the red, blue, and black bars represent significant differences for NS vs. NHS, NHS vs. PHS, and NS vs. PHS, respectively.

SPM analysis with repeated measures ANOVA revealed a significant difference between shoe conditions in GRF (Figure 2). Post-hoc analysis shows the NHS’s AP-GRF was smaller than NS at 77.1–90.3% and 94.6–100% of the stance phase (*p* < 0.001). The AP-GRF of NHS was smaller than PHS and was significant at 25–33.3%, 82.0–90.3%, and 94.6–100% of the stance phase (*p* < 0.001).

The post-hoc analysis results showed that the ML-GRF of NS was significantly larger than PNS during the stance phase (14.5–17.7%; 71.9–82.6%) (*p* < 0.001). At the 1.3–9.4% stance phases, the ML-GRF of NS was significantly more significant than the NHS (*p* < 0.001). At 1.3–4.6% and 8.8–10.3% of the stance phase, the ML-GRF PHS was significantly greater than NHS (*p* < 0.05).

The post-hoc analysis results showed that the vertical GRF of NHS in the third trimester of pregnancy was significantly larger than NS during the gait stance phase (91.8–100%) (*p* < 0.001). At 66.3–72.4% of the stance phase, PHS was significantly lower than the NS (*p* < 0.001). The vertical GRF of NHS was larger than that of PHS during 40–44%, 61–71.5%, and 92.5–100% of the stance phase (*p* < 0.001).

### 3.3. COP Trajectory

**Figure 3:**
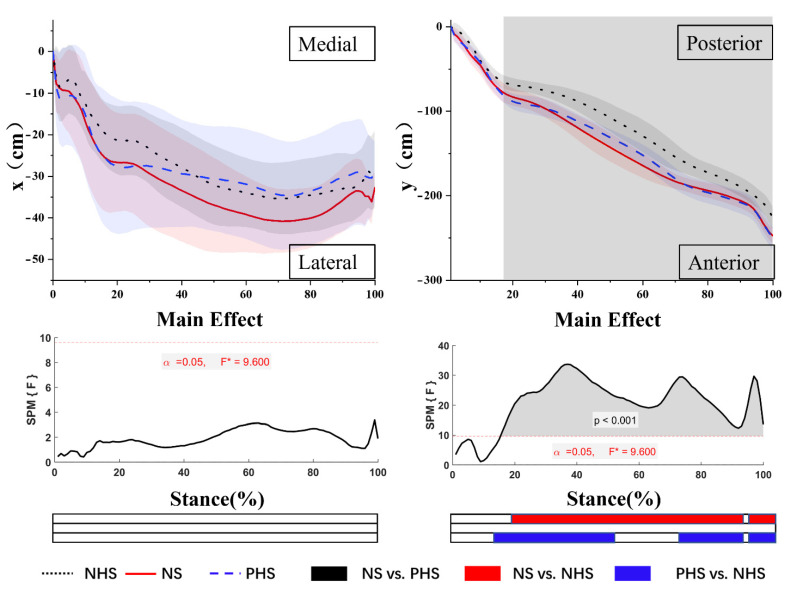
Mean cop trajectories in the x-time and y-time planes. The grey areas indicate significant differences between conditions, followed by the time-dependent F-values of the SPM. Colored bars beneath each plot indicate significant differences between waveforms, whereas the red, blue, and black bars represent significant differences for NS vs. NHS, NHS vs. PHS, and NS vs. PHS, respectively.

As shown in Figure 3, the results showed no difference in ML-COP between NHS, NS, and PHS. For AP-COP, there was a main effect. Post-hoc analysis showed that NHS demonstrated a significantly smaller range of AP-COP in NHS vs. NS for 19–90.5% and 93–100% of the stance phase. At 14.5–53.1%, 68.5–90% and 93.5–100% of the stance phase, the NHS posterior COP was significantly smaller than PHS (*p* < 0.05). 

### 3.4. COM-COP Inclination Angles

No significant differences were found in step peak medial angles and peak anterior angles. Significant main effects were found for peak posterior angles (Table 3). Post-hoc tests revealed significantly lower peak posterior angles for NHS compared with NS and PHS. 

## 4. Discussion

The primary purpose of this study was to investigate the differences in gait spatiotemporal parameters, 3D-GRF, and COP of the condition of NHS, NS, and PHS in the third trimester of pregnancy. Compared with PHS and NS, pregnant women wearing NHS showed a more stable gait posture in the anterior–posterior direction, with slower walking speed and smaller peak posterior COM-COP inclination angles.

### 4.1. Gait Spatiotemporal Parameters

Gait parameters changed with different heel heights of shoes [41]. Although studies have shown that 2/3 of falls during pregnancy occur due to smooth surfaces, sudden acceleration, or moving objects [6,8], gait changes caused by pregnancy are still one of the critical causes of falls in pregnant women [16]. Therefore, it is necessary to understand the influence of shoes with different heels on the gait spatiotemporal parameters of pregnant women in the third trimester.

This study found that participants wearing NHS showed decreased stride length and speed compared to PHS. Similar to our results, Benz (1998) reported that the NHS’s walking speed was significantly reduced due to a shorter stride length combined with an increased cadence [42]. Li et al. reported that walking with NHSs induced the upper body to tilt backward, which may have caused a disadvantage in the propulsion phase compared to walking with normal shoes [41]. This may be the reason for the decrease in stride length. PHS moved the center of gravity forward, and the forward tilt of the trunk assisted in moving the center of gravity outside the support area. There is more motivation during the duration of take-off [43,44], which may be the reason for the difference in stride length between NHS and PHS. Previous studies found that the habitual gait in the third trimester of pregnancy is characterized by slower speed and shorter step length, which may be caused by slow gait strategies [16]. Taking a shorter step during pregnancy reduced the gait’s energy consumption and increased the gait’s stability [16]. This change in stride length and speed may lead to changes in other gait parameters and may help increase gait stability in pregnant women. On the other hand, the decreased stride length may be due to unfamiliarity with NHS, which leads to anxiety about falls and a more conservative or unstable gait [16,45,46,47].

### 4.2. GRF

Ground reaction force (GRF), which can measure braking and propulsive forces during gait, is a summation of forces produced by all body segments [48]. Increases in magnitude and variability of the peaks of GRF during the weight acceptance and push-off phases are to be found in people with unstable locomotion [48]. Our result found that different heel-toe drops have no significant effect on the first and second peaks of vertical GRF. Therefore, it is reasonable to speculate that wearing NHS, NS, and PHS has little effect on the walking stability of pregnant women in the third trimester. 

The results showed no significant change in the ML-GRF during the stance phase, except in the early stance phase, the ML-GRF of NHS was significantly smaller than PHS and NS. Previous studies have shown that in the early stages of the stance phase (0–6% stance phase), the maximum ground reaction of the supporting foot is directed laterally and increases significantly with increasing walking speed [49]. This is similar to the results of our research. Our research results show that with the increase in heel-toe drop, the velocity also increases, which may be the reason for the difference in ML-GRF. Less energy is expended when the body is stable on the inside. Therefore, NHS has a smaller ML-GRF, which may be evidence of reduced energy consumption in pregnant women wearing NHS.

The AP-GRF included braking and propulsion peaks [50]. Our study found that the AP-GRF of the NHS propulsion peak was significantly smaller than NS and PHS. At present, there is controversy about the change in AP-GRF during pregnancy. Some researchers believe that there is no significant difference in AP-GRF during pregnancy, and other studies have shown that the AP-GRF decreases during pregnancy [51]. This may be due to edema of the pregnant foot during pregnancy, which interferes with flexion by increasing the width of the foot, resulting in reduced thrust. Our study found that it may be due to the thickness of the front palm of the NHS, which leads to disturbance of the flexion of the metatarsophalangeal joints, which may be the reason for the small AP-GRF during the propulsion phase.

### 4.3. COP Trajectory

COP is used to describe the complex dynamic functions of the foot and foot-ground interface during gait [52]. The COP is not only used as a dynamic stability index and measured risk or consequence of various lower limb musculoskeletal disorders [52,53,54,55]. The lack of lateral stability is known to be a risk factor for falls [52,55]. The results showed no significant difference in the range of ML-COP in NHS, NS, and PHS, which is consistent with the previous study [56]. No significant differences in the range and velocity of ML-COP were found in the flat shoes, medium heel lift shoes (16 mm), heel lift shoes (25 mm), and heel lift shoes (34 mm) [56]. NHS and PHS may not pose a more significant biomechanical challenge to the medial–lateral control.

The AP-COP displacement measures the fluency of the stance phase during regular gait, with higher AP-COP displacement and gait line length indicating a more physiological gait pattern [57,58]. The results showed that the AP-COP of NHS is significantly smaller than NS and PHS. Previous studies have shown that AP-COP moves forward and decreases during the stance phase in pregnant women [13,14]. Reduced COP displacement in the AP direction could be linked to the waddling type of gait adopted by pregnant women [13]. Raymaks et al. found that the AP-COP increases with the increase in heel height, the AP-COP of NS is significantly smaller than that of PHS, and leg muscle activation increases when walking in high heels [59]. The results of our study may indicate that women in the third trimester of pregnancy have the lowest degree of muscle activation when walking with NHS.

### 4.4. COM-COP Inclination Angles

The medial COM-COP inclination angle may be a sensitive measure of gait stability [37]. Our study found that the ML-ROM and the peak medial COM-COP inclination angles were not significantly different under different conditions, and we inferred that changing heel-toe drop within a certain range does not change the ML stability of pregnant women in the third trimester of pregnancy. Our findings indicate that heel-toe drop affects the peak posterior COM-COP inclination angle in pregnant women in the third trimester and that NHS is significantly smaller in the peak posterior COM-COP inclination angle than NS., which may benefit the stability during the propulsion phase. Previous studies on NHS showed that foot contact angle and the angle of the ankle NHS are significantly larger than those of NS. This may indicate that wearing NHS may benefit stability in the front and rear directions. Of course, this change may be related to the slower walking speed of NHS, which has been shown to affect gait changes and COM movement [60,61,62].

### 4.5. Limitations

There are still some limitations. The acute effect of the footwear conditions was investigated, and no conclusions can be drawn for longer-term or habituation effects. We only investigated the impact of three different heel-toe drops on gait parameters. The study sample will be expanded in the future, and electromyography (EMG) data will be included to infer further what mechanisms are involved in the generation and change of force. Future studies should explore the effects of long-term different heel-toe drops on gait in pregnant women in different periods and the longer-term effects.

## 5. Conclusions

This study compared the gait spatiotemporal parameters, GRF, and balance of pregnant women wearing different heel-toe drop shoes in the third trimester of pregnancy. The results are as follows: (1) NHS reduced the walking speed of women in the third trimester of pregnancy by reducing the stride. (2) The results showed that the impact of a heel-toe drop on the AP-GRF during the propulsion phase was relatively large, which might be due to the various dorsiflexion of the ankle with different heel-toe drop conditions. We inferred that changing heel-toe drop within a certain range does not change the ML stability of pregnant women in the third trimester of pregnancy. (3) We found that peak posterior COM-COP inclination angles are significantly smaller, so NHS may increase the stability of the pregnant women’s propulsion phase and help women maintain balance in the third trimester of pregnancy. Understanding the gait differences in NHS, NS, and PHS of pregnant women in the third trimester will provide information for future research, evidence for the design of shoes for pregnant women, and falls prevention.

## Figures and Tables

**Table 1:** List of abbreviations and acronyms used in this article.

Abbreviation	Explanation
PHS	Positive Heel Shoes
NHS	Negative Heel Shoes
NS	Normal Shoes
SPM	Statistical Parametric Mapping
ANOVA	Analyses of Variance
ACOG	American College of Obstetricians and Gynecologists
GRF	Ground Reaction Force
COP	Center of Pressure
AP	Anterior-Posterior
ML	Medial-Lateral

**Table 2:** Mean values, standard deviations, and results of the repeated measures ANOVA for spatiotemporal parameters.

Indexes (Unit)	NHS (Mean ± SD)	NS (Mean ± SD)	PHS (Mean ± SD)	F	*p*
Stride length (m)	0.99 ± 0.08 c	1.05 ± 0.07	1.11 ± 0.03 a	10.24	<0.001
Walking speed (m/s)	0.76 ± 0.11 bc	0.83 ± 0.16 a	0.90 ± 0.08 b	5.97	<0.001
Step frequency (step/s)	1.48 ± 0.13	1.56 ± 0.21	1.56 ± 0.14	1.51	0.25
Double support time/single support time (%)	0.32 ± 0.01	0.32 ± 0.02	0.31 ± 0.01	2.02	0.16

Note: NHS: negative heel shoes, NH: normal shoes, PHS: positive heel shoes. Post-hoc significant differences are marked with a (vs. NHS), b (vs. NS), c (vs. PHS).

**Table 3:** Mean values, standard deviations, and results of the repeated measures ANOVA for peak COM-COP inclination angle.

Indexes (Unit)	NHS (Mean ± SD)	NS (Mean ± SD)	PHS (Mean ± SD)	F	*p*
Peak medial angles (°)	3.28 ± 0.91	3.38 ± 0.70	3.14 ± 0.48	0.35	0.71
Peak anterior angles (°)	16.00 ± 1.79	15.90 ± 3.31	17.00 ± 1.61	1.22	0.30
Peak posterior angles (°)	12.82 ± 2.61 bc	15.22 ± 2.18 a	14.53 ± 1.72 a	16.52	<0.01

Note: NHS: negative heel shoes, NH: normal shoes, PHS: positive heel shoes. Post-hoc significant differences are marked with a (vs. NHS), b (vs. NS), and c (vs. PHS).

## Data Availability

The data that support the findings of this study are available on reasonable request from the corresponding author. The data is not publicly available due to privacy or ethical restrictions.
